# Biology and pathogenesis of *Acanthamoeba*

**DOI:** 10.1186/1756-3305-5-6

**Published:** 2012-01-10

**Authors:** Ruqaiyyah Siddiqui, Naveed Ahmed Khan

**Affiliations:** 1 The Aga Khan University, Karachi, Pakistan; 2 School of Veterinary Medicine and Science, University of Nottingham, England, UK

## Abstract

*Acanthamoeba *is a free-living protist pathogen, capable of causing a blinding keratitis and fatal granulomatous encephalitis. The factors that contribute to *Acanthamoeba *infections include parasite biology, genetic diversity, environmental spread and host susceptibility, and are highlighted together with potential therapeutic and preventative measures. The use of *Acanthamoeba *in the study of cellular differentiation mechanisms, motility and phagocytosis, bacterial pathogenesis and evolutionary processes makes it an attractive model organism. There is a significant emphasis on *Acanthamoeba *as a Trojan horse of other microbes including viral, bacterial, protists and yeast pathogens.

## Background

**Figure 1:**
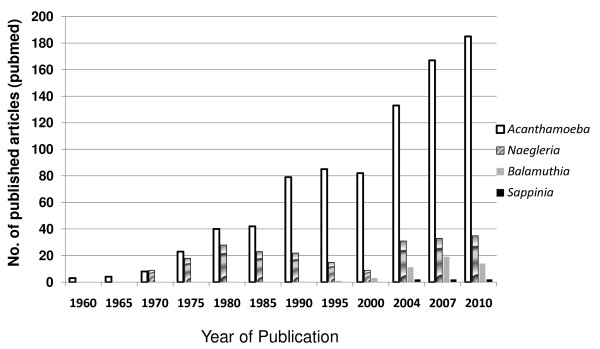
**Increasing scientific interest in the field of free-living amoebae as determined by published articles over the last five decades**. A pubmed search using "*Acanthamoeba*", "*Balamuthia*", *Naegleria*" or "*Sappinia*" was carried out.

*Acanthamoeba *is an opportunistic protist that is ubiquitously distributed in the environment. *Acanthamoeba *has two stages in its life cycle, an active trophozoite stage that exhibits vegetative growth and a dormant cyst stage with minimal metabolic activity. It is a causative agent of cutaneous lesions and sinus infections, vision-threatening keratitis and a rare but fatal encephalitis, known as granulomatous amoebic encephalitis [1-3]. The ability of *Acanthamoeba *to (i) produce serious human infections associated with a rise in the number of immunocompromised patients and contact lens wearers, (ii) their potential role in ecosystems, (iii) ability to act as a host/reservoir for microbial pathogens, and (iv) model organism for motility studies has led to a significant interest in this organism over the years (Figure 1). Furthermore, *Acanthamoeba *may have veterinary significance as demonstrated by the presence of amoebae in diseased or dead cows, dogs, pigs, rabbits, pigeons, sheep, reptiles, fish, turkeys, keel-billed toucan, *Ramphastos sulfuratus*, horses [4-6].

### Discovery of Amoebae

**Figure 2:**
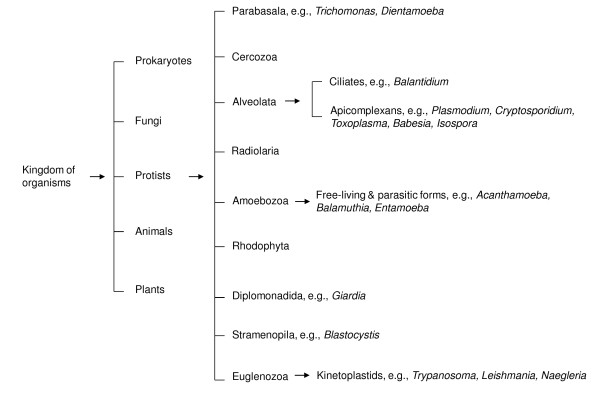
**The classification of protists, based on ribosomal rRNA sequences (modified from Khan NA *Acanthamoeba*: Biology and Pathogenesis, Caister Academic Press, 2009, ISBN: 978-1-904455-43-1)**.

Amoebae are among the earliest eukaryotes that have been studied since the discovery of the early microscope, e.g., *Amoeba proteus*, or closely related *Chaos *that is a genus of giant amoebae, varying from 1-5 mm in length. Based on rRNA sequences, it is estimated that amoebae have diverged from the main line of eukaryotic descent, sometimes between the divergence of yeast (~1.2 × 10^9 ^years ago) and the divergence of plants and animals (~1 × 10^9 ^years ago). Over the past several decades, these organisms have gained increasing attention due to their diverse roles in the ecosystem and in particular, their role in causing serious and sometimes fatal human infections (Figure 2).

• *Entamoeba histolytica *is a parasitic protist that was discovered in 1873 from a patient suffering from bloody dysentery [7,8] and named *E. histolytica *in 1903 [9,10]. This species was separated into one pathogenic (*E. histolytica*) and another non-pathogenic (*E. dispar*) [11], which also is capable of producing experimental lesions [12] and questioned by some authors if really it is unable to cause human disease [13].

• *Naegleria *is a free-living amoebae that was first discovered by Schardinger in 1899, who named it "*Amoeba gruberi*". In 1912, Alexeieff suggested its genus name as *Naegleria*, and much later in the 1970, Carter identified *Naegleria fowleri *as the causative agent of fatal human infections involving the central nervous system (CNS) [14].

• *Sappinia diploidea *is a free-living amoeba that was isolated from the faeces of lizards and from the soil in 1908-09, and then described as a causative agent of granulomatous amoebic encephalitis in 2001 [15].

• *Balamuthia mandrillaris *was discovered in 1986, from the brain of a baboon that died of meningoencephalitis and was described as a new genus, i.e., *Balamuthia *[3,16]. So far, only one species has been identified, *B. mandrillaris*. The majority of isolates have been isolated from necropsies while organic-rich soil has been suggested as a potential source. Like *Acanthamoeba*, it is known to produce infections of the central nervous system, lungs, sinuses and skin. Worryingly, granulomatous encephalitis due to *B. mandrillaris *has been reported in immunocompetent individuals indicating its potential threat to human and animal health.

• In 1930, *Acanthamoeba *was discovered as a contaminant of yeast culture, *Cryptococcus pararoseus *and was later placed in the genus *Acanthamoeba*, and then described as a causative agent of *Acanthamoeba *granulomatous encephalitis (AGE) in the 1960s and of keratitis in 1970s [17].

### Biology of *Acanthamoeba*

The term acanth (Greek "acanth" means "spikes") was added to "amoeba" to indicate the presence of spine-like structures (now known as acanthopodia) on its surface. It contains one or more prominent contractile vacuoles, whose function is to expel water for osmotic regulation [18]. Other types of vacuoles in the cytoplasm include lysosomes, digestive vacuoles and a large number of glycogen-containing vacuoles. The plasma membrane consists of proteins (33%), phospholipids (25%), sterols (13%), and lipophosphonoglycan (29%) [19,20]. The major phospholipids in *Acanthamoeba *are phosphatidylcholine (45%), phosphatidylethanolamine (33%), phosphatidylserine (10%), phosphoinositide (6%), and diphosphatidylglycerol (4%). The main fatty acids chains in *Acanthamoeba *are oleic acids (40-50%), and longer polyunsaturated fatty acids (20-30%) [21]. *Acanthamoeba *contains low levels of glycolipids. Glucose accounts for about 60% of the sugars of the glycolipids of the whole cells and of the plasma membranes. Among sterols, the non-saponifiable fraction of the total lipids extracted from the trophozoites of pathogenic *Acanthamoeba *possesses ergosterol and 7-dehydrostigmasterol [20]. *Acanthamoeba *has been shown to produce prostaglandins [22].

**Figure 3:**
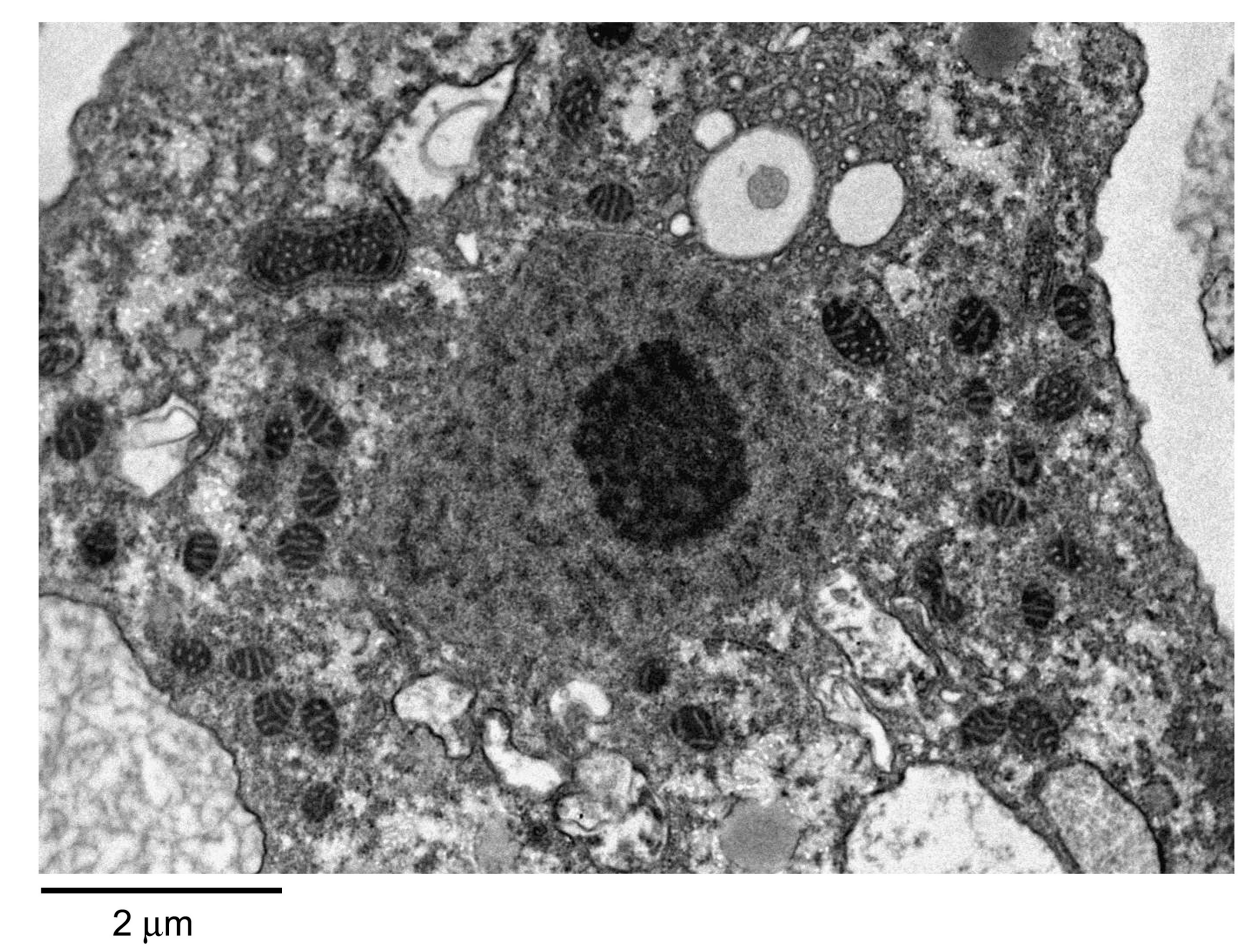
**The transmission electron micrograph of *Acanthamoeba *trophozoite**. M is mitochondria; N is nucleus; V is vacuole and arrow indicates plasma membrane.

*Acanthamoeba *trophozoite possesses large numbers of mitochondria (Figure 3). The genome size of mitochondrial DNA from *A. castellanii *belonging to T4 genotype is 41,591 bp [23]. *Acanthamoeba *normally possesses a single nucleus that is approximately one sixth the size of trophozoite (Figure 3), but multinucleate amoebae have been observed. The genome size of *A. castellanii *Neff strain, belonging to T4 genotype is approximately 45 Mb http://www.hgsc.bcm.tmc.edu/microbial-detail.xsp?project_id=163. Based on the coding sequence (CDS features, exon) analysis of 200 genes, it was calculated that there are on average 3 exons per gene (for comparison, *E. histolytica *possess 1.3 exons per gene, and *Dictyostelium discoideum *possess 2.3 exons per gene) [24].

*Acanthamoeba *has long been studied as a model eukaryotic cell with special emphasis on the actin cytoskeleton-based motility [25]. *Acanthamoeba *moves relatively fast compared to other cells, with a locomotory rate of approximately 0.8 μm/second. The movement involves the formation of a hyaline pseudopodium. The manner of *Acanthamoeba *movement is similar both at solid substrata and water-air interface. Adhesion forces developed between *Acanthamoeba *and the water-air interface are greater than gravity, and thus amoebae are also transported passively without detachment from the water surface [26]. Actin microfilaments are most concentrated just beneath the plasma membrane, and are responsible for resisting tension and forming cytoplasmic protrusions.

### Life cycle of *Acanthamoeba*

**Figure 4:**
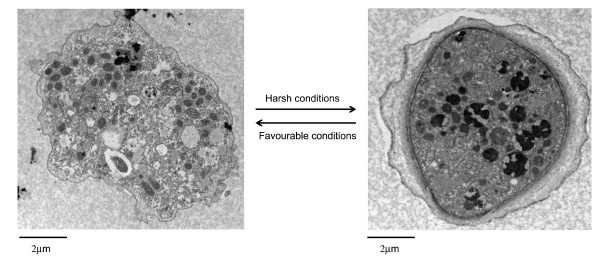
**The life cycle of *Acanthamoeba *spp. Under favourable conditions, *Acanthamoeba *remains in the trophozoite form and divides mitotically (**A**) and produces infection, while under harsh conditions amoeba transforms into a dormant cyst form (**B**) that is highly resistant to harsh conditions**.

*Acanthamoeba *has two stages in its life cycle, a vegetative trophozoite stage with a diameter of 13-23 μm and dormant cyst stage of 13-23 μm (Figure 4). During the trophozoite stage (Greek "tropho" means "to nourish"), *Acanthamoeba *feeds on organic particles as well as other microbes and divides mitotically under optimal conditions (food supply, neutral pH, ~30°C) and 50-80mOsmol [27]. Exposure to harsh conditions result in cellular differentiation into a double-walled cyst form [28]. The outer walls consists of proteins and polysaccharides, while the inner wall possesses cellulose [29-31]. Both walls are normally separated by a space, except at certain points where they form opercula in the centre of ostioles (exit points for excysting trophozoite). The cyst wall composition for *A. castellanii *belonging to T4 genotype has been shown to contain 33% protein, 4 - 6% lipid, 35% carbohydrates (mostly cellulose), 8% ash, and 20% unidentified materials [29-31]. Using gas chromatography combined with mass spectrometry, the carbohydrate composition of cyst walls revealed a high percentage of galactose and glucose and small amounts of mannose and xylose [32]. Linkage analysis revealed several types of glycosidic linkages including the 1,4-linked glucosyl conformation indicative of cellulose (Table 1).

**Table 1:** Glycosyl linkage analysis of *Acanthamoeba castellanii *cyst wall saccharides (reproduced with permission from Dudley *et al.*, 2009).

**Glycosyl Residue**^**1**^	**Area**^**2**^	Percentage Present
Terminal Mannopyranose	563313	3.2

5 linked Xylofuranose/4XylP	1236950	7.0

3 linked Galactopyranose	5038076	28.6

4 linked Glucopyranose	3908275	22.2

3,4 linked Galactopyranose	2392068	13.6

3,4 linked Glucopyranose	1063116	6.0

2,4 linked Gluco or Galactopyranose	783793	4.4

4,6 linked Mannopyranose	1368136	7.8

3,6 Linked Galactopyranose	1273911	7.2

^1^Note that 5 linked xylofuranose and 4-linked xylopyranose are not distinguished in this assay as they have the same derivative. ^2^Area percentages are not corrected for response factor and thus may not be representative of molar ratios.

### Distribution in the environment and clinical settings

*Acanthamoeba *has been isolated from diverse natural environments including sea water, ocean sediments, beaches, pond water, soil, fresh water lakes, hot spring resorts, salt water lakes, Antarctica, water-air interface, and even from the air. They have been isolated from bottled mineral water, distilled water bottles, thermally-polluted factory discharges, cooling towers of the electric and nuclear power plants, Jacuzzi tubs, ventilation ducts, humidifiers, air-conditioning units, shower heads, kitchen sprayers, sewage, compost, vegetables, surgical instruments, contact lenses and their cases, pigeon droppings, fresh water fish, as well as other healthy, diseased, and dead animals. They have been recovered from hospitals, physiotherapeutical swimming pools, dialysis units, portable and stationary eye wash stations, human nasal cavities, throat, pharyngeal swabs, lung tissues, skin lesions, human faeces, corneal biopsies, maxillary sinus, mandibular autografts, stool samples, urine of critically ill patients, cerebrospinal fluids and the brain necropsies. Based on the above, it is accepted that *Acanthamoeba *is ubiquitously present in the environment and that we commonly encounter this organism in our routine lives as evidenced by the presence of anti-*Acanthamoeba *antibodies in up to 100% healthy populations in New Zealand and more than 85% in individuals of London that came from different countries [33,34].

### Role in the Ecosystem

In soil, protists such as amoebae, flagellates and ciliates have two major ecological roles: (i) influencing the structure of the microbial community, and (ii) enhancing nutrient recycling. Both of these activities are associated with soil protists feeding on bacteria thus regulating bacterial populations in the soil. Among protists, free-living amoebae are the dominant bacterial consumers and are responsible for up to 60% of the total reduction in bacterial population [35]. The primary decomposers (bacteria) directly decompose organic materials but are inefficient in releasing minerals from their own mass. The secondary decomposers, such as free-living amoebae, consume the primary decomposers and release mineral nutrients as waste products that are tied up in the primary decomposer's biomass. In this way, protists such as *Acanthamoeba *(as well as other grazers) make nutrients available that would otherwise remain inaccessible for much longer. The soil containing *Acanthamoeba *and bacteria showed significantly greater mineralization of carbon, nitrogen, and phosphorous compared with the soil containing bacteria but without *Acanthamoeba *[36,37]. As well as bacterial consumption, amoebae promote bacterial populations in the soil. The mineral regeneration by the secondary decomposers (protists such as amoebae), relieved nutrient limitation for the primary decomposers. This was demonstrated with the findings that when nitrogen was limiting (but carbon present), nitrogen mineralization by *Acanthamoeba *permitted continued growth of bacteria (*Pseudomonas paucimobilis*) resulting in a greater bacterial biomass [36,37]. And when carbon was limiting, *Acanthamoeba *was almost entirely responsible for nitrogen mineralization, with bacteria (*Pseudomonas paucimobilis*) contributing little. Using an experimental model system, the effects of grazing by *Acanthamoeba *on the composition of bacterial communities in the rhizosphere of *Arabidopsis thaliana *demonstrated reduction in bacterial populations leading to positive effect on plant growth [35-37]. Overall, *Acanthamoeba *appears to play an important role in the regulation of bacterial populations in the environment and the nutrient cycling, thus contributing to the functioning of the ecosystems.

### Genotyping

Based on rRNA gene sequences, the genus *Acanthamoeba *is divided into 17 different genotypes to date (T1 - T17) (Table 2) [38-41]. Each genotype exhibits 5% or more sequence divergence between different genotypes. The majority of human infections due to *Acanthamoeba *have been associated with the isolates of the T4 genotype. For example, more than 90% of *Acanthamoeba *keratitis (AK) cases have been linked with this genotype. Similarly, T4 has been the major genotype associated with the non-keratitis infections such as AGE and cutaneous infections. At present, it is unclear why T4 isolates are most abundant in human infections but it is likely due to their greater virulence and properties that enhance their transmissibility as well as their reduced susceptibility to chemotherapeutic agents. Future studies will identify virulence traits and genetic markers limited only to certain genotypes, which may help clarify these issues. A current list of genotypes and their association with the human infections is presented in Table 2.

**Table 2:** Known *Acanthamoeba *genotypes and their associations with human diseases, i.e., keratitis and granulomatous encephalitis.

*Acanthamoeba *genotypes	Human disease association
**T1**	Encephalitis

**^T2a**	Keratitis, Encephalitis

**^T2b - ccap1501/3c-alike sequences**	NA

**T3**	Keratitis

T4*	Keratitis, Encephalitis

**T5**	Keratitis, Encephalitis

**T6**	Keratitis

**T7**	NA

**T8**	NA

**T9**	NA

**T10**	Keratitis, Encephalitis

**T11**	Keratitis

**T12**	Encephalitis

**T13**	NA

**T14**	NA

**T15**	Keratitis

**T16**	NA

**T17**	NA

*this genotype has been most associated with both diseases

^basis of T2 division into T2a and T2b has been proposed by Maghsood *et al.*, (2005)

NA - no disease association has been found yet

### *Acanthamoeba *keratitis

**Figure 5:**
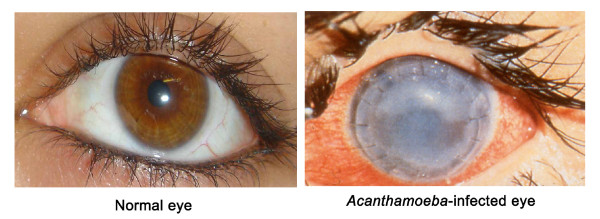
**(A) Normal eye and (B) Infected eye exhibiting recurrent *Acanthamoeba *infection following corneal transplant with severe corneal damage and loss of vision**.

Although it can occur in non-contact lens wearers, it is mostly associated with the use of contact lenses. Overall this is a multifactorial process involving (i) contact lens wear for extended periods of time, (ii) lack of personal hygiene, (iii) inappropriate cleaning of contact lenses, (iv) biofilm formation on contact lenses, and (v) exposure to contaminated water [3]. The sequence of events in AK involves breakdown of the epithelial barrier, stromal invasion by amoebae, keratocyte depletion, induction of an intense inflammatory response, photophobia and finally stromal necrosis with blinding consequences (Figure 5) [42,43]. Recent studies have reported a significant increase in the number of AK patients in the USA, Australia, Italy, New Zealand, and Brazil [44-48]. This is further supported with 2 recent outbreaks of AK where a dramatic rise was seen in tertiary care centers in Singapore and the United States [49]. At present there are more than 120 million people wearing contact lenses, throughout the world, thus there is a growing need to be aware of the associated risks. This is particularly important in view of the ineffectiveness of cleaning solutions of some contact lens products. For example in 2006, Bausch & Lomb (USA) voluntarily withdrew their contact lens solution "ReNu with MoistureLoc contact lens solution" from the market. This was a result of an outbreak of eye infection in the contact lens wearers http://www.drugattorneys.com/fda-reports/fusariumkeratitis-051906.cfm resulting in hundreds of lawsuits being brought against Bausch & Lomb. The company recognized the problem and removed all ReNu with MoistureLoc products worldwide. This is not a one-off. Since the discovery of the first corneal lenses in 1949, there have been several outbreaks of contact lens-associated infections throughout the world with microbial ones being the most serious ones. Time and time again, the negligence of many manufacturers has been highlighted. For example in 2005, Dr. Epstein alerted Bausch & Lomb of the ineffectiveness of their ReNu with MoistureLoc contact lens solution to kill *Fusarium*, a claim that was rejected by the manufacturer. Again, in 2007, the Centers for Disease Control and Prevention (CDC) issued a public health alert about an increased AK risk. This outbreak was linked primarily to Complete Moisture Plus No-Rub contact lens solution. The manufacturer, Advanced Medical Optics (AMO), had voluntarily recalled the solution and was encouraging consumers not to use it until further information was available. Overall, there is a clear need to be aware of the associated risks of the contact lens use, particularly in developing countries, where health surveillance may not be appropriate.

### Diagnosis

The diagnosis of AK is problematic and it is often misdiagnosed as bacterial, viral or fungal keratitis. The use of contact lenses by the patient together with excruciating pain is strongly indicative of this infection. The use of *in vivo *confocal microscopy has emerged as a valuable non-invasive tool for the clinical diagnosis in severe infectious keratitis with high sensitivity [50,51]. The confirmatory evidence comes from demonstrating parasites using laboratory-based assays. The cultivation of *Acanthamoeba *from the corneal biopsy or from contact lenses/cases remains the most widely used assay. Immunofluorescence assays and multiplex real-time PCR methods [52] have also been developed. The multiplex assay is of value for the simultaneous detection of pathogenic free-living amoebae in the same sample. The use of real-time fast-duplex TaqMan PCR for the simultaneous detection of 10 different genotypes of *Acanthamoeba *can detect 0.1 cyst/μl [53]. In addition, matrix-assisted laser desorption-ionization time-of-flight mass spectrometry and ^1^H NMR spectroscopy has been shown to be of potential value in the rapid identification of *Acanthamoeba *in the clinical specimens.

### Treatment

Early diagnosis followed by aggressive treatment is essential for the successful prognosis. No single agent is shown to be uniformly effective against all isolates/genotypes of *Acanthamoeba*. Multiple factors including varied clinical presentation and virulence of *Acanthamoeba *account for a lack of correlation between *in vitro *activity and *in vivo *efficacy. The treatment regimen includes polyhexamethylene biguanide or chlorhexidine digluconate together with propamidine isethionate or hexamidine, is effective. If bacteria are also associated with the infection, addition of antibiotics, i.e., neomycin or chloramphenicol is recommended [54].

### *Acanthamoeba *granulomatous encephalitis

AGE is a rare infection but it almost always proves fatal. It is of major concern in view of increasing numbers of immunocompromised patients who are susceptible hosts, individuals undergoing immunosuppressive therapy and excessive use of steroids. Individuals with lymphoproliferative or hematologic disorders, diabetes mellitus, pneumonitis, renal failure, liver cirrhosis or other hepatic diseases, gamma-globulinaemia or patients undergoing organ/tissue transplantation with immunosuppressive therapy, steroids and excessive antibiotics are at risk [55,56]. The gross pathology of the autopsied brains show severe edema and haemorrhagic necrosis. The microscopic findings of the post-mortem necropsies reveal amoebae cysts, predominantly in the perivascular spaces in the parenchyma indicating involvement of the cerebral capillaries as the sites of amoebae entry into the CNS. It is widely accepted that the route of entry for *Acanthamoeba *include the respiratory tract leading to amoebae invasion of the alveolar blood vessels, followed by the haematogenous spread. *Acanthamoeba *entry into the CNS most likely occurs through the blood-brain barrier [55,56]. As AGE is a secondary infection, it is difficult to determine its true burden on human health. The approximate rate of AGE-associated deaths has been suggested as 1.57 deaths per 10,000 HIV/AIDS deaths [3].

### Diagnosis

The symptoms of AGE are similar to other CNS infections including virus, bacteria and fungi. The neurological manifestations of AGE may vary and include headache, fever, behavioral changes, hemiparesis, lethargy, stiff neck, agitation, aphasia, ataxia, vomiting, nausea, cranial nerve palsies, increased intracranial pressure, seizures, and coma [55,56]. The magnetic resonance imaging or computerized tomography of the brain shows ring-enhancing lesions exhibiting a single or multiple space-occupying mass in the cerebral cortex but severely immunocompromised patients may not exhibit such lesions. The CSF findings shows pleocytosis with lymphocytic predominance, increased protein concentrations, decreased glucose concentrations and minimal cloudiness [2], however the CSF may be devoid of cells in HIV-positive patients. High levels of *Acanthamoeba*-specific antibodies in patient's serum is indicative of AGE infection. The antibody levels in normal populations may be in the range of 1:20 to 1:60 [33,57] but patients with severely impaired immune system may not develop a high titre. The confirmatory evidence comes from demonstration of amoebae in the infected tissues.

### Treatment

There is no recommended treatment and the majority of cases are diagnosed at the post-mortem stage. A lack of available antiamoebic compounds together with selectivity of the blood-brain barrier has led to more than 90% mortality rate. Few successful cases involved the use of ketoconazole, fluconazole, sulfadiazine, pentamidine isethionate, amphotericin B, azithromycin, itraconazole, or rifampicin [1,58,59] but overall the prognosis remains poor.

### Pathogenesis

Parasite adhesion to the host cell is a primary step and is mediated by a 130 kDa mannose-binding protein (MBP) expressed on the surface of *Acanthamoeba *[60]. *Acanthamoeba mbp *consists of 6 exons and 5 introns that spans 3.6 kbp. The 2.5 kbp cDNA codes for an 833 amino acids precursor protein with a signal sequence (residues 1-21aa), an *N*-terminal extracellular domain (residues 22-733aa) with five *N*- and three *O*-glycosylation sites, a transmembrane domain (residues 734-755aa), and a *C*-terminal intracellular domain (residues 756-833aa). Other adhesins include a laminin-binding protein with a predicted molecular mass of a 28.2 kDa, a 55 kDa laminin-binding protein and a > 207 kDa adhesin [61,62]. The initial binding leads to secondary events such as phagocytosis and toxin production resulting in the host cell death in a phosphotidylinositol 3-kinasedependent (PI3K) manner [63]. The downstream effectors of PI3K involves activation of proapoptotic molecules, Bak and Bax, loss of mitochondrial membrane potential and release of cytochrome *c *as well as caspase activation, all well-known mediators of the apoptosis [64-66]. Among host cell receptors, Toll-like receptor 4 (TLR4) showed involvement in *Acanthamoeba *recognition and exerting an effect through adaptor protein, Myeloid differentiation primary response 88 that led to the activation of transcription factors, nuclear factor-kappa B signalling through extracellular signal-regulated kinases (ERKs) inducing the secretion of cytokines including interleukin-8, tumor necrosis factor-alpha and interferon-beta in human corneal cells [67]. Using human brain microvascular endothelial cells (HBMEC), which constitute the blood-brain barrier, it is shown that *Acanthamoeba *abolished the HBMEC transendothelial electrical resistance by degrading occludin and zonula occludens-1 tight junction proteins in a Rho kinase-dependent manner leading to increased permeability [68].

**Figure 6:**
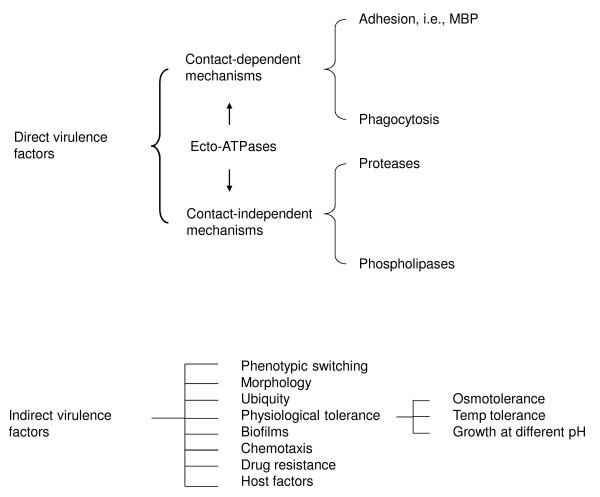
***Acanthamoeba *pathogenesis involves contact-dependent and -independent factors together with indirect virulence features (taken from Khan NA *Acanthamoeba*: Biology and Pathogenesis, Caister Academic Press, 2009, ISBN: 978-1-904455-43-1)**.

Other factors that may contribute to *Acanthamoeba *pathogenesis include ecto-ATPases of approximate molecular weights of 62, 100, 218, 272, > 300 kDa [66] and these are involved in caspase-3 activation (Figure 6) [69]. The neuraminidase activities of *Acanthamoeba *could be relevant in the colonization of the parasite, and also important in producing damage of the sialic acid-rich corneal epithelium and in the alterations of glycolipids associated with meningoencephalitis. Interestingly, the neuraminidases of *Trypanosoma cruzi *and *Acanthamoeba *are immunologically related as demonstrated by antibodies against neuraminidase of *Trypanosoma cruzi*, which reacted with *Acanthamoeba *[70]. Two superoxide dismutases have been identified in *Acanthamoeba*: an iron superoxide dismutase (~50 kDa) and a copper-zinc superoxide dismutase (~38 kDa). The superoxide dismutase catalyzes the dismutation of the superoxide into oxygen and hydrogen peroxide and play a role in antioxidant defence. These enzymes may provide additional targets for chemotherapy and immuno-diagnosis of *Acanthamoeba *infections. *Acanthamoeba *has been shown to display plasminogen activator activity by catalyzing the cleavage of host plasminogen to form plasmin, which can activate host matrix metalloproteinases leading to degradation of the basement membranes.

Of contact-independent factors, *Acanthamoeba *possess hydrolytic enzymes including elastases [71], phospholipases [72], glycosidases and a variety of serine, cysteine and metalloproteases (Figure 6) [2,3,42,43]. However, their precise mechanisms of action at the molecular-level are only beginning to emerge. That some of the above proteases are secreted only by the clinical isolates may indicate their role as potent virulence factors and/or diagnostic targets. Future studies in the role of proteases as vaccine targets, search for novel inhibitors by screening of chemical libraries, or rational development of drugs based on structural studies will enhance our ability to target this pathogen. Overall, the mechanism by which *Acanthamoeba *breaches biological barriers is complex and is likely to involve both parasite (adhesins, proteases, phospholipases) as well as host determinants [interleukin-beta, interleukin-alpha, tumor necrosis factor- alpha, interferon-gamma, host cell apoptosis]. In addition to aforementioned potential virulence factors, the ability of *Acanthamoeba *to survive harsh environmental conditions and its resistance to chemotherapeutic drugs by differentiating into cysts contributes to its pathogenicity.

### Immune response to *Acanthamoeba *infections

The recurrence of AK infections is common, suggesting that the corneal infection alone does not induce protective immunity against the parasite antigens. Experimental animals immunized orally with *Acanthamoeba *antigens mixed with the cholera toxin showed significantly lower infection rates compared with the control groups (21.4% *versus *72.6% respectively) and protection was associated with higher levels of parasite-specific sIgA. More specifically, oral immunization using recombinant MBP improved AK and protection was associated with the presence of elevated levels of anti-MBP sIgA in tears of the immunized animals [73]. Similarly, oral immunization with a serine protease (~133 kDa) reduced the severity of the corneal infection by modulating MMP-2 and MMP-3 expression [74]. Overall, it is suggested that AK patients show decreased overall levels of sIgA as well as specific anti-*Acanthamoeba *sIgA, however the role of sIgA was questioned in a recent study in which neither normal tears nor AK tears had any protective effects on *Acanthamoeba*-mediated corneal epithelial cell cytotoxicity. Tear factors, in addition to sIgA such as lysozyme, lactoferrin, beta-lysins, prostoglandins, and other compounds with antimicrobial and immunological properties were also shown to have no significant effects on *Acanthamoeba*-mediated binding to and cytotoxicity of human corneal epithelial cells [75]. Tears also contain complement that is composed of serum-borne molecules in a cascade-like manner. *Acanthamoeba *directly activates the complement system via the alternative pathway, however pathogenic amoebae are resistant to complement-mediated lysis due to expression of complement regulatory proteins including decay accelerating factor [76]. The presence of macrophages in corneas exposed to the parasite-laden contact lenses prevented the development of full-blown AK *in vivo *by inducing an inflammatory response, in particular secretion of macrophage inflammatory protein-2 [77].

For AGE, immunization with *Acanthamoeba *antigens using intranasal, intraperitoneal, intravenous or oral routes of administration had a protective effect validating that AGE is limited to individuals with a weakened immune response. The complement pathway and antibodies in the presence of phagocytes show potent lytic activity against *Acanthamoeba *in a contact-dependent manner. These interactions also stimulate secretion of pro-inflammatory cytokines including interleukin-1-beta, interleukin-6 and tumor necrosis factor-alpha [76-79]. Other studies in mice have shown significant increased natural killer cell activities in *Acanthamoeba*-infected animals suggesting that natural killer cells may also play a role in the protective immunity [80]. Overall, a debilitated immune status of the host is a pre-requisite in AGE but the underlying mechanisms together with the role of the host ethnic origin (i.e., genetic predisposition) remain incompletely understood. Pathogenic *Acanthamoeba *are shown to degrade chemokines and cytokines, antibodies, complement pathway, and macrophages [76,81,82].

### Future prospects for treatment

A murine monoclonal anti-idiotypic antibody and a synthetic killer mimotope (mimics a yeast killer toxin) showed broad spectrum anti-amoebic activities suggesting their potential use in the prevention and therapy of *Acanthamoeba *infections [83]. The Fab fragment of a monoclonal antibody specifically reactive to *A. castellanii *cell surface was covalently linked to the A chain of diphtheria toxin [84]. This immunotoxin inhibited cell division completely, suggesting that specific antibodies coupled with cytotoxic agents could be a useful method in the development of therapeutic interventions or preventative measures. To enhance the potency of available drugs, propamidine isethionate combined with dimethylsulfoxide proved to be highly effective suggesting that use of a carrier for known anti-amoebic drugs may increase their penetration into the cyst form of the organism, which is normally refractory to drug treatment. To this end, the use of liposomes has been shown to improve the potency of pentamidine isethionate *in vitro *[85]. Similarly, the use of chitosan microspheres improved *in vitro *anti-amoebic activity of rokitamycin. Such methods will be useful in transporting the drug for either ocular application to treat AK or nasal administration as an alternative route for the administration of the drug to the brain in AGE therapy. Small interfering RNAs (siRNAs) against the catalytic domains of extracellular serine proteases and glycogen phosphorylase showed promise in the rational development of therapeutic interventions [86]. Photodynamic chemotherapy by linking amoeba-specific antibodies with photosensitizers such as phthalocyanine or Hypocrellins B may be advantageous over conventional methods due to its localized use, in particular for eye infections. In addition, the programmed cell death in protists has emerged as a fascinating field of parasite biology and could serve as a basis of novel anti-Acanthamoebic drugs [87-89].

### *Acanthamoeba*: Trojan Horse of the Microbial World

The majority of *Acanthamoeba *isolates harbor endosymbionts which may include viruses, yeast, protists and bacteria, some of which are potential human pathogens. The exact nature of symbiosis and the benefit they represent for the amoeba host are unknown. It is suggested that such interactions may help transmit microbial endosymbionts to the susceptible hosts and/or endosymbionts may contribute to the pathogenicity of *Acanthamoeba *[3,90]. Future studies in the identification of virulence factors of the endosymbiont and of the host, and their precise role in disease will clarify these issues.

*Acanthamoeba *is known to host the largest known virus, Mimivirus, initially mistaken for a parasitic bacterium with a particle size of 400 nm and genome size of 1.2 million bp [91]. Among 911 protein coding genes, 10% exhibit similarity to proteins of known functions blurring the established boundaries between viruses and Archea/Bacteria, a finding that may have huge implications in our understanding of the evolutionary processes [3,91].

*Acanthamoeba *is shown to harbour a variety of viruses including coxsackieviruses, adenoviruses, poliovirus, echovirus, enterovirus, or vesicular stomatitis virus, and yeast, *Cryptococcus neoformans*, *Blastomyces dermatitidis*, *Sporothrix schenckii*, *Histoplasma capsulatum*, *Streptomyces californicus *and *Exophiala dermatitidis*, and protists including *Cryptosporidium *and *Toxoplasma gondii *[3,91].

Among bacterial pathogens, *Acanthamoeba *are shown to host/reservoir for *Aeromonas *spp., *Bacillus cereus*, *Bartonella *spp., *Burkholderia *spp., *Burkholderia pickettii*, *Campylobacter jejuni*, *Candidatus Odyssella thessalonicensis*, *Chlamydophila pneumoniae*, *Coxiella burnetii*, *Cytophaga *spp., *Escherichia coli *O157, neuropathogenic *Escherichia coli *K1, *Flavobacterium *spp., *Francisella tularensis*, *Helicobacter pylori*, *Legionella pneumophila*, *Listeria monocytogenes*, *Staphylococcus aureus*, Methicillin-resistant *Staphylococcus aureus*, *Mycobacteria tuberculosis*, *M. avium*, *M. leprae*, *Parachlamydia Acanthamoebae*, *Pasteurella multocida*, *Prevotella intermedia*, *Porphyromonas gingivalis*, *Pseudomonas aeruginosa*, *Rickettsia*, *Salmonella typhimurium*, *Shigella dysenteriae*, *S. sonnei*, *Simkania negevensis*, *Vibrio cholerae*, *V. parahaemolyticus*, *Waddlia chondrophila *as well as novel bacterial endosymbionts that are related to *Caedibacter caryophilus*, *Holospora elegans *and *Holospora obtuse*, which were proposed as '*Candidatus Caedibacter Acanthamoeba*e', '*Candidatus Paracaedibacter Acanthamoeba*e' and '*Candidatus Paracaedibacter symbiosus*' suggesting the usefulness of amoeba co-culture to recover novel chlamydial strains [3,91]. With the remarkable implications of parasite-parasite interactions, which may contribute to the evolution of one (either bacteria or *Acanthamoeba*) or both parasites to become successful human and animal pathogens and transmission of microbial pathogens in the environment, this area of research is of particular significance.

## Conclusions

*Acanthamoeba *has gained increasing attention from the scientific community studying cellular microbiology, environmental biology, physiology, cellular interactions, molecular biology, biochemistry and the evolutionary studies. This is due to their versatile roles in the ecosystem and their ability to capture prey by phagocytosis (similar to macrophages), act as vectors, reservoirs and as a Trojan horse for microbial pathogens, and to produce serious human infections including a blinding keratitis and fatal encephalitis. This unicellular organism has been used extensively to understand the molecular biology of cell motility. Being a eukaryote, *Acanthamoeba *presents an excellent model for cell differentiation studies. The recent availability of the *Acanthamoeba *genome, together with the development of transfection assays and the RNA interference methods [86] will undoubtedly increase the pace of our understanding of this complex but fascinating organism.

## Competing interests

The authors declare that they have no competing interests.

## Authors' contributions

NK conceived the study. RS and NK wrote the original manuscript. All authors approved the final manuscript.
